# A mechanistic view of long noncoding RNAs in cancer

**DOI:** 10.1002/wrna.1699

**Published:** 2021-10-19

**Authors:** Lauren Winkler, Nadya Dimitrova

**Affiliations:** ^1^ Department of Molecular, Cellular, and Developmental Biology Yale University New Haven Connecticut USA

**Keywords:** cancer, genetic models, long noncoding RNAs, mechanism

## Abstract

Long noncoding RNAs (lncRNAs) have emerged as important modulators of a wide range of biological processes in normal and disease states. In particular, lncRNAs have garnered significant interest as novel players in the molecular pathology of cancer, spurring efforts to define the functions, and elucidate the mechanisms through which cancer‐associated lncRNAs operate. In this review, we discuss the prevalent mechanisms employed by lncRNAs, with a critical assessment of the methodologies used to determine each molecular function. We survey the abilities of cancer‐associated lncRNAs to enact diverse *trans* functions throughout the nucleus and in the cytoplasm and examine the local roles of *cis*‐acting lncRNAs in modulating the expression of neighboring genes. In linking lncRNA functions and mechanisms to their roles in cancer biology, we contend that a detailed molecular understanding of lncRNA functionality is key to elucidating their contributions to tumorigenesis and to unlocking their therapeutic potential.

This article is categorized under:

Regulatory RNAs/RNAi/Riboswitches > Regulatory RNAs
RNA in Disease and Development > RNA in Disease

## Graphical abstract

Long noncoding RNAs (lncRNAs) are increasingly recognized as functional mediators of cellular processes in both homeostasis and disease. The observation that lncRNAs are frequently dysregulated in cancer has sparked new efforts to understand the functions and mechanisms by which lncRNAs contribute to tumorigenesis.

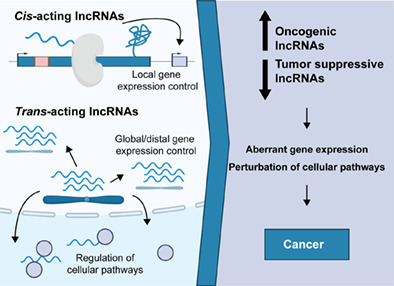

## 1 INTRODUCTION

Cancer is traditionally viewed as a complex disease arising from the gradual accumulation of genetic and epigenetic alterations in protein‐coding genes (Hanahan & Weinberg, 2011). Over the past decades, detailed mechanistic studies of cancer‐associated proteins have linked specific protein aberrations to the acquisition of cancer hallmarks and enabled the development of targeted therapies for previously intractable cancers. The discovery that over 70% of the human genome is transcribed to yield hundreds of thousands of noncoding RNAs has transformed our view of the functional genomic space (Bertone et al., 2004; Carninci et al., 2005; Djebali et al., 2012; Iyer et al., 2015; Kapranov et al., 2007). A growing number of studies have since sought to determine the contributions of this expansive noncoding transcriptome to tumorigenesis.

Gene expression profiling by RNA sequencing (RNAseq) has revealed that a subset of noncoding RNAs, termed long noncoding RNAs (lncRNAs) on account of their length of more than 200 nucleotides, are frequently differentially expressed in tumor samples compared to normal tissues (Yan et al., 2015). Importantly, the increased or decreased expression of some lncRNAs has been found to strongly correlate with cancer progression and patient prognosis. In fact, a survey of lncRNA expression patterns across different cancer types has suggested that the deregulation of lncRNA expression may exhibit a higher specificity to cancer type and grade compared to changes in the expression of messenger RNAs (mRNAs; Yan et al., 2015). These correlative findings have provided initial indications that lncRNAs may represent an unexplored reservoir of diagnostic and prognostic markers in cancer (Arun et al., 2018).

The identification of recurrent cancer‐associated genetic aberrations in several lncRNA‐producing loci has raised the possibility that lncRNAs may be drivers, and not simply passengers, of cancer development (Beroukhim et al., 2010; Prensner & Chinnaiyan, 2011; Rheinbay et al., 2020). Efforts to model the recurrent genetic alterations of lncRNAs in cellular and organismal models of cancer have provided evidence that some lncRNAs are functional mediators of cancer‐relevant processes (Olivero & Dimitrova, 2020). These studies have revealed that genetic aberrations in lncRNA loci can contribute to the acquisition of cancer hallmarks, such as hyperproliferation, enhanced survival, altered metabolism, and increased metastatic dissemination (Huarte, 2015). LncRNAs have also been reported to support the development of drug resistance and immune evasion (Bester et al., 2018; Joung et al., 2017). These examples have suggested that lncRNAs have the capacity to directly modulate diverse aspects of tumorigenesis.

Despite a growing appreciation of the diverse roles of cancer‐associated lncRNAs, their mechanisms and functional elements remain poorly characterized. To date, only a handful of studies have examined the sequence and structural basis for lncRNA activity. The scarcity of mechanistic insights has limited our understanding of the contributions of lncRNAs to cancer development and impeded efforts to exploit their therapeutic potential. Here, we describe the current paradigms for the molecular activities of lncRNAs. By highlighting the experimental strategies used to characterize individual lncRNAs, we aim to define the relationship between lncRNA mechanisms and cellular and molecular pathologies in cancer. We propose that an integrative approach is essential for the validation of lncRNAs as drivers and therapeutic targets in cancer and other diseases.

## 2 EXPERIMENTAL STRATEGIES FOR STUDYING LNCRNA MECHANISMS

**FIGURE 1:**
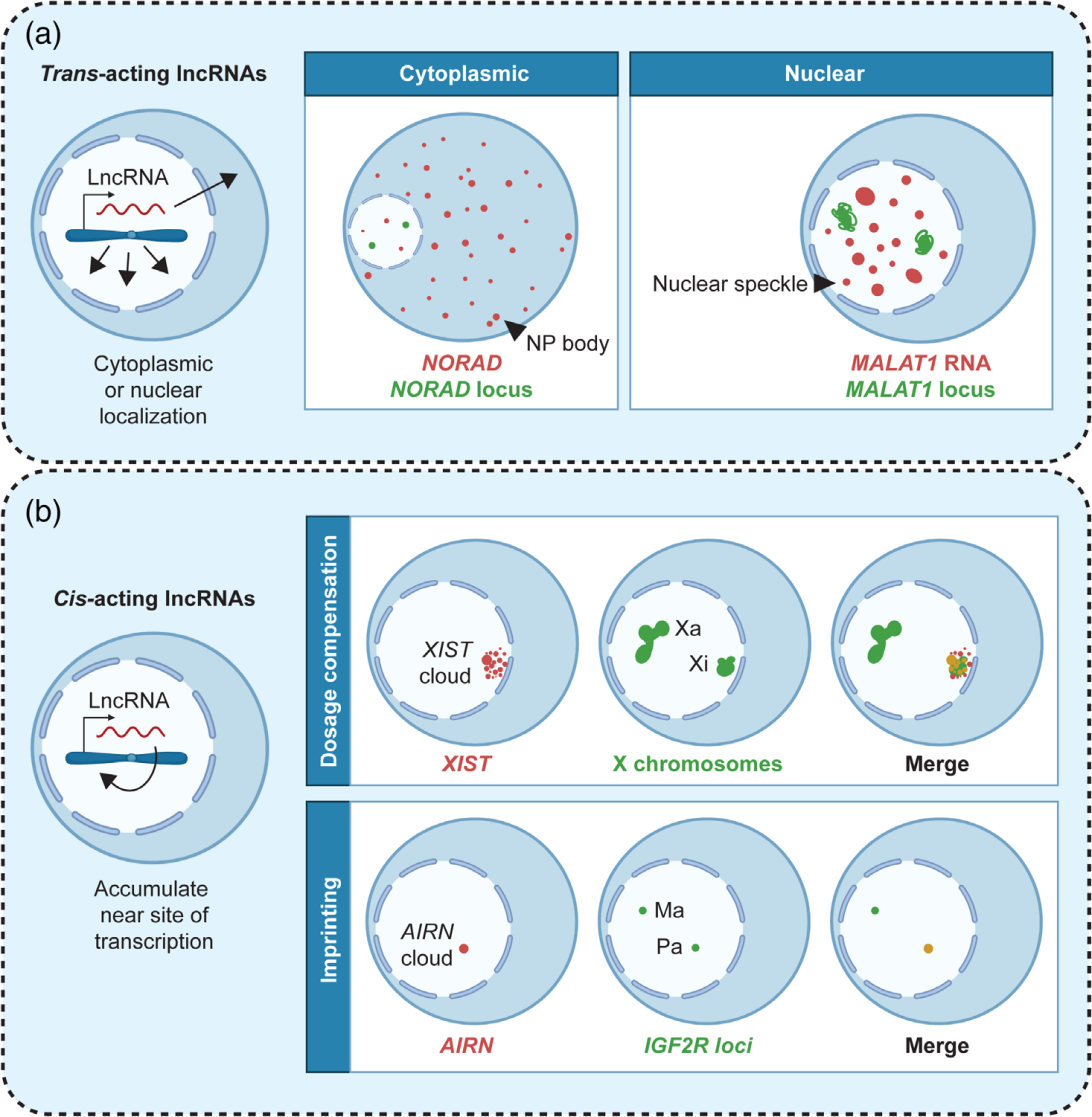
Categorizing lncRNAs based on subcellular localization and site of action. (a) *Left—*following transcription, *trans*‐acting lncRNA localize to distant sites within the nucleus and/or are exported to the cytoplasm; *right—NORAD* largely localizes to the cytoplasm, where it interacts with PUM proteins to form phase‐separated condensates known as NP bodies (Elguindy & Mendell, 2021; Lee et al., 2016), while *MALAT1* remains within the nucleus and localizes to nuclear speckles (Hutchinson et al., 2007); (b) *Left*—*cis*‐acting lncRNAs accumulate at or near their site of transcription. *Right*—*XIST* (red) is expressed from and spreads on the inactive (Xi) but not the active (Xa) X chromosome (green) in female mammalian cells. The lncRNA *AIRN (*red) is expressed from and represses the expression of genes from the imprinted *IGF2R* locus (green) on the paternal (Pa) but not maternal (Ma) allele

The identification of as many as 100,000 lncRNAs in mammalian genomes has opened the possibility that these newly discovered RNA molecules may harbor a wide range of novel functional and mechanistic features, akin to proteins (Derrien et al., 2012; Iyer et al., 2015). However, the observation that lncRNAs are characterized by relatively poor evolutionary conservation at the sequence level has impeded initial efforts to define conserved functional elements (Cabili et al., 2011; Ulitsky, 2016). Instead, it has been hypothesized that lncRNAs may act through the formation of intricate intramolecular secondary and tertiary structures capable of interfacing with DNA, RNA, and proteins (Guttman & Rinn, 2012). In the context of this mechanistic paradigm, *trans*‐acting lncRNAs have been proposed to play diverse regulatory roles throughout the nucleus and in the cytoplasm (Kopp & Mendell, 2018; Rinn & Chang, 2012; Figure 1a). Examples of *trans*‐acting lncRNAs include non‐coding RNA activated by DNA damage (*NORAD*) and metastasis‐associated lung adenocarcinoma transcript 1 (*MALAT1*), which are two scaffold RNAs that act in the context of cytoplasmic NP (NORAD‐PUM) bodies (Elguindy & Mendell, 2021; Lee et al., 2016; Tichon et al., 2016) and nuclear speckles (Tripathi et al., 2010), respectively (Figure 1a). *Cis*‐acting lncRNAs, on the other hand, have been defined based on their accumulation near their sites of transcription and their roles in the local regulation of gene expression (Gil & Ulitsky, 2020; Kopp & Mendell, 2018; Figure 1b). Well‐established examples of repressive *cis*‐regulatory lncRNAs include X‐linked lncRNAs with essential roles in X chromosome inactivation, such as X inactive specific transcript (*XIST*; Sahakyan et al., 2018), and lncRNAs expressed from imprinted loci, such as antisense of *IGF2R* nonprotein coding RNA (*AIRN*; Nagano et al., 2008) and *KCNQ1* overlapping transcript 1 (*KCNQ1OT1*; Mohammad et al., 2008; Pandey et al., 2008; Figure 1b). Beyond dosage compensation, antisense noncoding RNA in the *INK4* locus (*ANRIL*; Yap et al., 2010), myeloid RNA regulator of Bim‐induced death (*Morrbid*; Kotzin et al., 2016), and plasmacytoma variant translocation 1, isoform b (*Pvt1b*; Olivero et al., 2020) have been proposed to repress the expression of their neighboring genes. On the other hand, *HOXA* distal transcript antisense RNA (*HOTTIP*; Wang et al., 2011), *LincRNA‐p21* (Dimitrova et al., 2014), and *LincRNA‐Cox2* (Carpenter et al., 2013) have been implicated in local gene expression activation.

**FIGURE 2:**
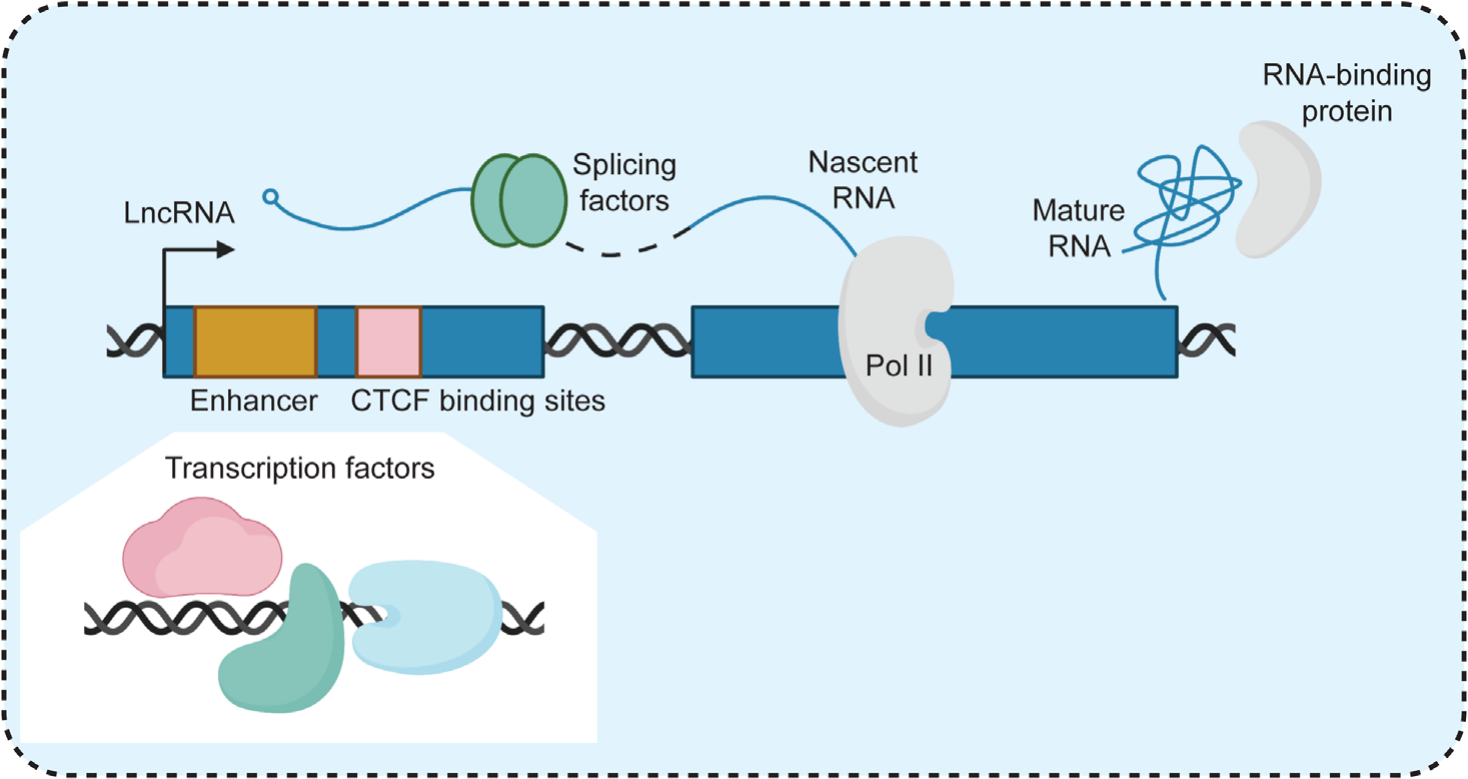
Layers of overlapping functional elements in *cis*‐regulatory loci. *Cis*‐acting lncRNA‐producing loci may act through one or more tightly coupled mechanisms. DNA elements such as promoters, enhancers, and CTCF binding sites may bind transcription factors that directly regulate local gene expression (Alexanian et al., 2017; Paralkar et al., 2016). Alternatively, the transcriptional process, including associated phenomena such as splicing, may enact *cis*‐regulation through transcriptional interference or by modulating the local transcriptional and epigenetic landscape (Allou et al., 2021; Engreitz et al., 2016; Latos et al., 2012). Finally, sequence and structural features of the lncRNA itself may enable it to interact with proteins that activate or repress local gene expression (Pandey et al., 2008; Pandya‐Jones et al., 2020)

Notably, simply because a lncRNA is transcribed, it does not necessarily mean that it produces a functional molecule. Recent studies provide evidence that functional lncRNA loci can act through sequence‐ or even transcription‐independent mechanisms (Espinosa, 2016) (Figure 2). On the one hand, several studies described lncRNA‐producing loci that can modulate the epigenetic and transcriptional landscape of nearby genes through the act of transcription (Anderson et al., 2016; Engreitz et al., 2016; Isoda et al., 2017; Latos et al., 2012). On the other hand, a different set of studies determined that the lncRNA transcripts may in fact be nonfunctional byproducts of underlying DNA regulatory elements such as enhancers (Engreitz et al., 2016; Paralkar et al., 2016). These observations have raised questions about the functional significance of specific features of lncRNA transcripts, such as RNA sequences or structural motifs. In this section, we briefly describe the common genetic, molecular, and biochemical tools used to elucidate the mechanistic basis of lncRNA activities, with a specific focus on the unique experimental and conceptual challenges that emerge when characterizing *cis*‐acting lncRNAs (Bassett et al., 2014; Kopp & Mendell, 2018).

### 2.1 Distinguishing between *trans*‐ and *cis*‐acting lncRNAs

**FIGURE 3:**
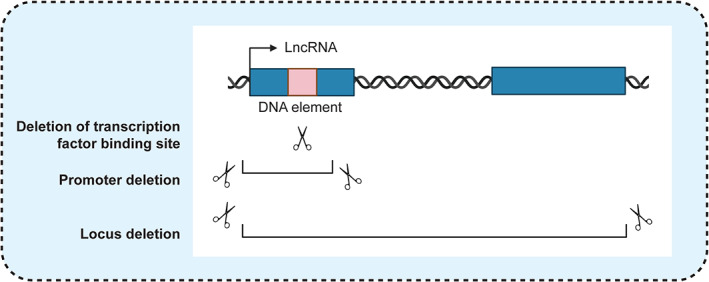
Inhibition of lncRNAs through genetic deletion approaches. Large deletions, such as ones that entail the excision of a locus or promoter, abolish lncRNA expression but can also grossly affect chromatin architecture and/or inadvertently delete functional DNA elements. Mutagenesis of DNA regulatory motifs, such as transcription factor binding sites, introduces finer genetic modifications but cannot dissociate between functional RNA and DNA elements

A combination of loss‐of‐function experiments, gene expression profiling, and subcellular localization studies has been applied to differentiate between *trans*‐ and *cis*‐acting lncRNAs. In loss‐of‐function studies, transient downregulation has been successfully achieved for many lncRNAs using antisense oligonucleotide (ASO) gapmer‐mediated knockdown or RNA interference (RNAi), although the mechanism by which RNAi mediates the degradation of nuclear and chromatin‐associated lncRNAs has remained unclear (Lennox & Behlke, 2016; Stojic et al., 2018). In parallel, studies have accomplished stable inhibition of target lncRNAs through genetic deletion approaches, such as promoter or locus deletions in cell lines and animal models (Bassett et al., 2014). While deletions effectively abolish lncRNA expression, even minimal genetic perturbations have the potential to destroy functional regulatory DNA sequences, such as transcription factor or CTCF binding sites (Bassett et al., 2014) (Figure 3). To overcome this caveat, alternative genetic approaches have aimed to insert transcriptional terminators to suppress transcription from endogenous lncRNA loci (Kopp & Mendell, 2018). This has been achieved through the knock‐in of polyadenylation signals (PAS) or transcriptional STOP cassettes to induce premature transcriptional termination.

Following lncRNA inhibition, targeted quantitative real‐time PCR (qRT‐PCR) analysis or unbiased RNAseq profiling have been used to determine whether a given lncRNA acts locally to regulate the expression of its neighboring genes or has global regulatory functions. Notably, inhibition of many *cis*‐acting lncRNAs has been found to produce relatively mild effects on the expression of neighboring genes (Gil & Ulitsky, 2018). This observation has suggested that many *cis*‐acting lncRNAs are primarily fine‐tuners of gene expression, promoting or suppressing the expression of target genes past or below a functional threshold, respectively (Dimitrova et al., 2014; Olivero et al., 2020). On the other hand, global gene expression changes following lncRNA inhibition should be interpreted cautiously as *trans* effects can, in some cases, be explained through *cis*‐regulatory functions. As an example, promoter deletion of the lncRNA *LincRNA‐p21* leads to a global suppression of Polycomb target genes but these gene expression changes are an indirect consequence of the role of *LincRNA‐p21* as a local transcriptional activator of the cyclin‐dependent kinase inhibitor 1a, also known as *p21* (*Cdkn1a/p21*; Dimitrova et al., 2014).

The key to differentiating *cis* versus *trans* activities has been determining whether exogenously introduced transcripts can rescue lncRNA function in loss‐of‐function experiments. Ectopic constructs are expected to recapitulate the function of *trans*‐acting but not *cis*‐acting lncRNAs, since the latter would not localize to the endogenous lncRNA locus. In the case of the *cis*‐regulatory *LincRNA‐p21*, exogenous overexpression of *LincRNA‐p21* in *LincRNA‐p21*‐deficient cells does not rescue *Cdkn1a/p21* or Polycomb target expression levels (Dimitrova et al., 2014). Similarly, the introduction of *Pvt1b* from an ectopic construct does not recapitulate transcriptional repression of *Myc* (Myelocytomasis), while overexpression of *Pvt1b* from the endogenous locus following CRISPR activation (CRISPRa) is able to suppress *Myc* (Olivero et al., 2020). An exception to this rule is the X‐inactivation lncRNA *Jpx*, which can activate *Xist* expression through both *cis* and *trans* mechanisms in a dose‐dependent manner, likely through its ability to act as a decoy and sequester negative regulators of *Xist* both locally and globally (Carmona et al., 2018; Sun et al., 2013; Tian et al., 2010).

Determining the localization of a lncRNA by subcellular fractionation or fluorescence in situ hybridization has also provided direct clues to whether a lncRNA may function in *cis* or in *trans*. For example, subcellular fractionation and RNA FISH visualization of *cis*‐regulatory lncRNAs, such as *XIST* and lncRNAs from imprinted loci, have revealed their close association with chromatin, while *trans*‐acting lncRNA, such as *MALAT1* and *NORAD1*, have been detected in the nucleoplasmic and cytoplasmic fractions, respectively (Elguindy et al., 2019; Tripathi et al., 2010). The recent development of single‐molecule RNA fluorescence in situ hybridization (smRNA‐FISH) has transformed the field by allowing the visualization of individual lncRNA molecules, including lowly expressed lncRNAs (Cabili et al., 2015; Raj et al., 2008; Raj & Rinn, 2019). smRNA‐FISH has been successfully used to establish the accumulation of functional intergenic RNA repeat element *(FIRRE)*, *LincRNA‐p21*, and *Pvt1b* at their sites of transcription by co‐localizing exon‐ and intron‐specific probes (Dimitrova et al., 2014; Hacisuleyman et al., 2014; Olivero et al., 2020). In a different instance, immunofluorescence (IF)/smRNA‐FISH combined with high‐resolution microscopy has revealed detailed mechanistic insights, such as the key role of the central region of the nuclear paraspeckle assembly transcript 1 (*NEAT1*) RNA in organizing structural proteins within the core of nuclear paraspeckles (Yamazaki et al., 2018).

### 2.2 Identification of binding partners of lncRNAs

Many lncRNAs have been proposed to operate through their interactions with DNA, RNA, and proteins. Thus, experimental identification of lncRNA interaction partners has been key to the mechanistic characterization of both *trans*‐ and *cis*‐acting lncRNAs. Recently, a number of techniques have been developed for the unbiased identification of lncRNA‐interaction partners. Approaches such as chromatin isolation by RNA purification (ChIRP) and capture hybridization analysis of RNA targets (CHART) have been used to map the genomic regions occupied by a lncRNA of interest (Chu et al., 2011; Simon et al., 2011). These techniques have provided valuable information about the genomic targets of the structural *trans*‐acting lncRNAs *NEAT1*, *MALAT1*, and *FIRRE* (Hacisuleyman et al., 2014; West et al., 2014). On the other hand, RNA and DNA split‐pool recognition of interactions by tag extension (RD‐SPRITE), developed to comprehensively examine RNA–DNA and RNA–RNA interactions, has revealed the widespread roles of *cis*‐acting lncRNAs in forming stable nuclear compartments in spatial proximity to their transcriptional loci. An additional suite of techniques has focused on the detection of lncRNA–RNA interactions (Engreitz et al., 2014; Lu et al., 2016). For example, RNA antisense purification (RAP) has been used to systematically map RNA–RNA interactions (RAP‐RNA) and to facilitate the identification of RNAs that interact with a lncRNA of interest (Engreitz et al., 2014). Alternatively, techniques such as psoralen analysis of RNA interactions and structures (PARIS) can be used to study entire RNA interactomes (Lu et al., 2016). Importantly, these and similar approaches also detect intramolecular base‐pairing interactions, and therefore have the potential to yield further insights into functional secondary structures within a lncRNA of interest. Finally, biochemical tools have been successfully used to identify proteins that bind lncRNAs directly or as part of a multi‐factor complex. A series of approaches based on cross‐linking, followed by pull‐down of RNA species with labeled probes, and mass spectrometric identification of associated proteins have been developed to identify lncRNA‐binding proteins. In the case of *XIST*, three independent strategies led to the identification of over 80 *XIST* binding proteins (Chu et al., 2015; McHugh et al., 2015; Minajigi et al., 2015). Subsequent studies validated many of these interactions and elucidated how they contribute to the process of X chromosome inactivation (Brockdorff et al., 2020). On the other hand, *NORAD* was found to specifically associate with the Pumilio (PUM) proteins PUM1 and PUM2 through a series of 15 repetitive Pumilio response elements (PREs; Lee et al., 2016; Tichon et al., 2018). An emerging theme from these studies has been the potential role of lncRNAs–protein interactions in nucleating phase‐separated compartments with unique biophysical and molecular characteristics, such as the *XIST* cloud (Pandya‐Jones et al., 2020), *NEAT1*‐containing paraspeckles (Yamazaki et al., 2018), and *NORAD*‐dependent NP bodies (Elguindy & Mendell, 2021).

### 2.3 Unique challenges with the functional dissection of lncRNA‐producing *cis*‐regulatory loci

**FIGURE 4:**
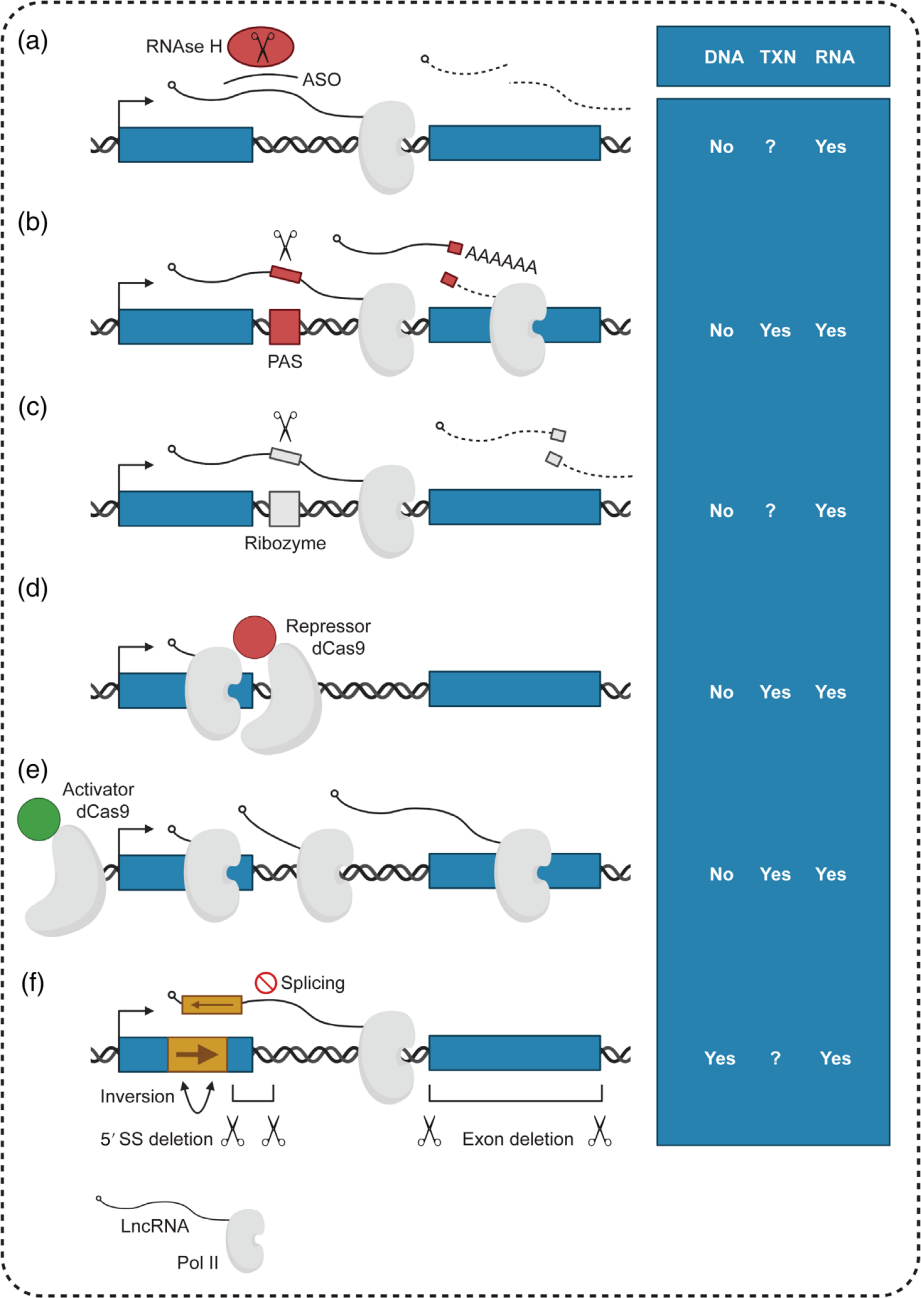
Experimental approaches for dissecting the activity of *cis*‐regulatory lncRNAs. *Left—*(a) ASO gapmers act co‐ or post‐transcriptionally to trigger RNAse H‐dependent degradation of target RNAs (Lai et al., 2020; Lee & Mendell, 2020); (b,c) Insertion of short genetic elements such as a PAS or self‐cleaving ribozyme permits the stable, specific knockdown of host lncRNAs (Engreitz et al., 2016; Latos et al., 2012; Sleutels et al., 2002; Tuck et al., 2018); (d,e) CRISPRi, based on recruitment of catalytically inactive dead Cas9 (dCas9), may disrupt transcription by sterically blocking transcriptional elongation by Pol II (Dahlman et al., 2015). This inhibitory effect can be augmented by fusing Cas9 to repressive chromatin‐modifying proteins such as the KRAB domain (Gilbert et al., 2013). Analogously, CRISPRa, exploits the ability of dCas9 to target fused activating domains to promoters, thereby boosting transcription (Gilbert et al., 2014); (f) Various genetic approaches, such as sequence inversion (Mohammad et al., 2008), 5′ splice site (SS) or downstream exon deletions (Allou et al., 2021; Engreitz et al., 2016) have been used to investigate the importance of specific sequence elements. *Right*—Table indicates whether the approach is expected to affect DNA elements (DNA), act of transcription (TXN), or the RNA molecule (RNA); “Yes”, the element is affected, “No”, the element is not affected, and “?” the effects are unknown or context‐specific

While initial studies proposed that many *cis*‐acting lncRNAs may act as scaffolds that guide the recruitment of protein‐binding partners to specific genomic locations (Rinn & Chang, 2012), subsequent models have questioned the role of the mature RNA molecule in *cis*‐regulation. A major challenge to the functional characterization of *cis*‐acting lncRNAs has been the difficulty of experimentally dissociating the mature transcript from the transcriptional process or the underlying DNA regulatory elements within the locus (Bassett et al., 2014; Kopp & Mendell, 2018; Figure 4). While commonly used for mRNAs and *trans*‐acting lncRNAs, RNAi knockdown of chromatin‐bound lncRNAs is expected to be ineffective. As an alternative, ASO‐mediated degradation by RNAse H acts co‐transcriptionally in the nucleus to efficiently deplete *cis*‐acting lncRNAs (Figure 4a). However, recent studies have suggested that ASOs might also disrupt the act of transcription (Lai et al., 2020; Lee & Mendell, 2020).

As previously discussed, genetic deletion of a lncRNA‐associated regulatory element, promoter, or locus effectively abolishes the production of the lncRNA molecule but also affects the act of transcription and perturbs the DNA sequence (Bassett et al., 2014; Figure 3). Vice versa, amplification of entire loci leads to increased lncRNA expression but also increases the copy number of *cis*‐acting DNA regulatory sequences, such as enhancers (Tseng et al., 2014). To overcome some of the limitations, studies have employed PAS or STOP cassette insertions in endogenous lncRNA loci to disrupt transcription without removing underlying DNA elements (Allou et al., 2021; Anderson et al., 2016; Engreitz et al., 2016; Latos et al., 2012; Sleutels et al., 2002; Figure 4b). These approaches have been used to determine that the length of transcription is key to the function of some *cis*‐regulatory lncRNAs, such as lncRNAs from imprinted loci. Weaknesses of these approaches include the observation that the insertion of a single PAS, while convenient from a genome editing perspective, can be inefficient in the context of longer or highly transcribed lncRNAs (Engreitz et al., 2016). Moreover, PAS‐mediated termination still allows the production of nascent transcripts, which may include key functional elements. While more efficient in terminating transcription, larger STOP cassettes, which usually contain multiple polyadenylation sequences and a selection marker, may have additional consequences such as altering the chromatin organization of the locus.

As an emerging alternative, the insertion of self‐cleaving ribozymes in lncRNA transcripts has been used to achieve transcript‐specific knockdown (Tuck & Bühler, 2021). Self‐cleaving ribozymes are naturally occurring bacterial and viral RNA elements, such as the Hammerhead ribozyme and the Hepatitis Delta virus ribozyme, with short sequences capable of forming tertiary structures that undergo a self‐cleavage reaction (Tang & Breaker, 2000). When inserted into a lncRNA, self‐cleaving ribozymes have been shown to induce the cleavage and subsequent degradation of the host transcript (Camblong et al., 2009; Figure 4c). Further high‐throughput efforts have been dedicated to engineering these ribozymes into drug‐responsive devices (Xiang et al., 2019). While this line of approaches has been hailed as the first genetic tool to effectively dissociate the mature RNA molecule from the act of transcription, a number of additional questions remain to be addressed. For example, it is not clear to what extent the sequence and local structural context at the insertion site may impact ribozyme activity (Tuck et al., 2018). Moreover, it is not clear how the insertion of various self‐cleaving ribozymes affects the production and processing of nascent and mature lncRNA transcript (Fong et al., 2009).

Clustered regularly interspaced short palindromic repeats (CRISPR)‐based tools for epigenetic control have recently gained popularity as they have the unique capability to modulate the expression of lncRNAs from their endogenous loci and are therefore particularly useful for the dissection of the functions of *cis*‐regulatory lncRNAs (Gilbert et al., 2014; Figure 4d,e). CRISPR inhibition (CRISPRi) can disrupt transcription by sterically blocking elongating RNA Polymerase II (Pol II) or by recruiting repressive chromatin‐modifying enzymes, such as Kruppel associated box (KRAB), to the lncRNA promoter (Gilbert et al., 2013; Liu et al., 2020). CRISPR activation (CRISPRa), on the other hand, can activate the expression of lncRNAs through the local recruitment of activating factors, such as p65 and heat shock transcription factor 1 (HSF1) in CRISPR‐synergistic activation mediator (CRISPR‐SAM; Bester et al., 2018; Dahlman et al., 2015). One CRISPRi screen highlighted the *cis*‐repressive activities of the promoter of the lncRNA *Pvt1* on the expression of the Myc oncogene (Cho et al., 2018). Combined CRISPRi and CRISPRa approaches were analogously used to determine that production of the p53‐regulated lncRNA *LincRNA‐Gadd45γ* is both necessary and sufficient for the activation of its neighbor, *Gadd45γ*, during the cellular response to stress (Tesfaye et al., 2021). On the other hand, CRISPR‐based technologies have opened some unique challenges, including the potential of CRISPRi and CRISPRa factors to affect locations that are genomically distant but physically proximal to the promoter of the target lncRNA due to the three‐dimensional chromatin organization (Thakore et al., 2015).

Additional targeted genetic perturbations of various RNA features have also informed on the diverse functional features of *cis*‐acting lncRNAs (Figure 4f). For example, deletion of the first 5′ splice site in the lncRNA *Blustr* was found to recapitulate the effects of *Blustr* promoter deletion and PAS insertion on the nearby Scm like with four MBT domains 2 (*Sfmbt2*) gene, suggesting that promoter‐proximal splicing is critical for *cis*‐regulation by the *Blustr* locus (Engreitz et al., 2016). In contrast, deletion of three downstream exons had no effect on *Sfmbt2* levels, indicating that the full‐length lncRNA sequence is dispensable (Engreitz et al., 2016). In contrast, both deletion and inversion of a region of *KCNQ1OT1* relieved the repression of nearby imprinted genes, suggesting a role for production of the mature lncRNA (Mohammad et al., 2008, 2010). Taken together, these examples illustrate the power of complementary genetic approaches to provide important insights into the mechanisms of *cis*‐regulation.

Crucially, for all of these approaches, the inability to experimentally rescue the deficiency of *cis*‐acting lncRNAs with exogenously expressed constructs has made it difficult to control for potential off‐targets.

## 3 MECHANISMS OF CANCER‐ASSOCIATED LNCRNAS

### 3.1 *Trans*‐acting lncRNAs in cancer

#### 3.1.1 Global epigenetic and transcriptomic deregulation by lncRNAs overexpressed in cancer

**FIGURE 5:**
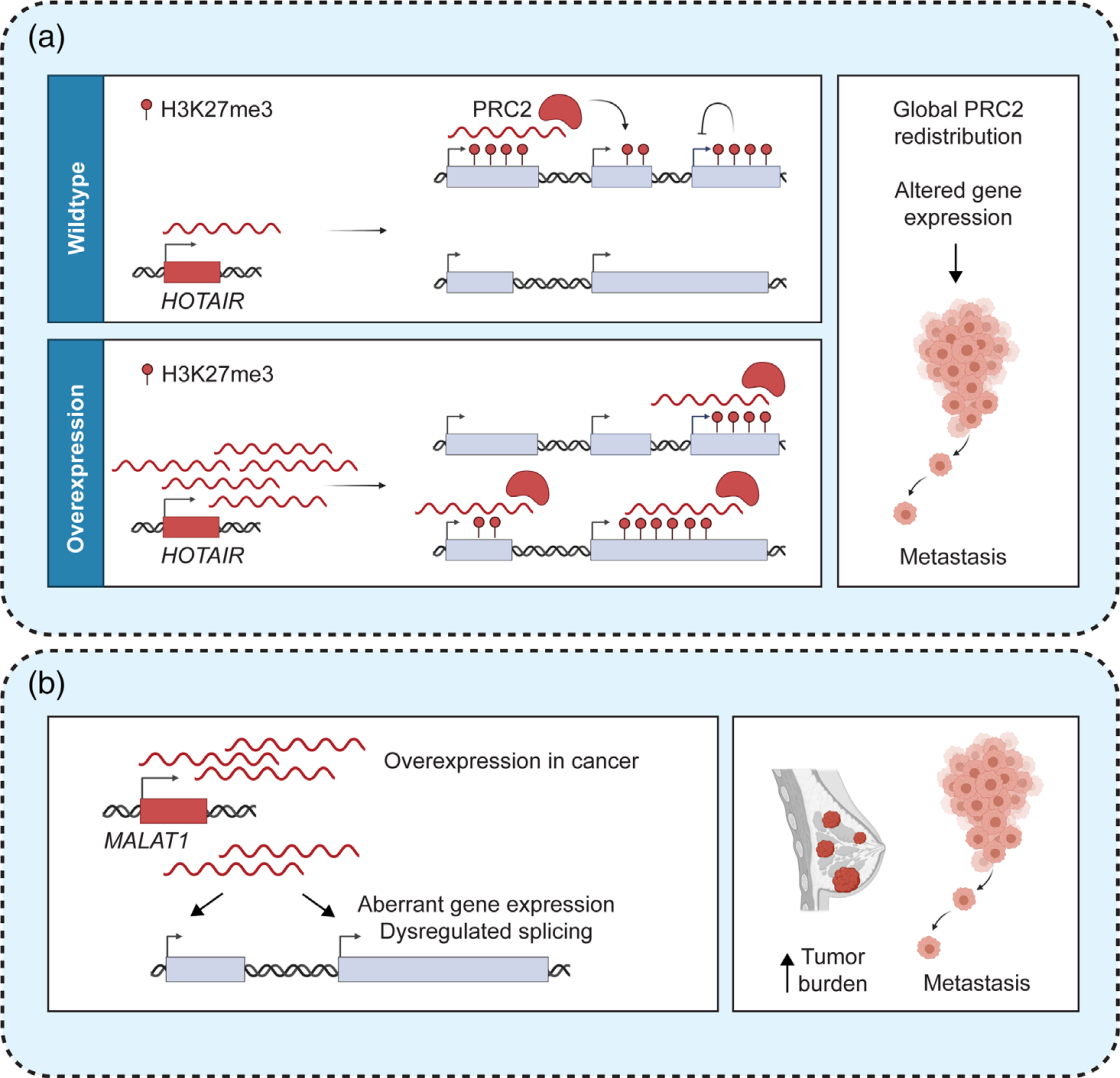
Overexpression of lncRNAs promote cancer progression through global epigenetic and transcriptional reprogramming. (a) *HOTAIR* overexpression leads to global PRC2 retargeting, leading to H3K27me3 deposition and downregulation of noncanonical PRC2 target genes with roles in metastasis suppression (Gupta et al., 2010); (b) *MALAT1* overexpression promotes cellular dedifferentiation, increased tumor burden, and metastatic dissemination through an unknown mechanism

Knowledge of the physiological functions and mechanisms of a lncRNA can reveal how deregulation of its expression in cancer cells may facilitate tumorigenesis. For example, the lncRNA *HOTAIR* (HOX antisense intergenic RNA) is overexpressed in primary and metastatic breast cancer, and there is a strong correlation between increased *HOTAIR* expression and the development of aggressive disease (Gupta et al., 2010). Mechanistically, *HOTAIR* was the first lncRNA implicated in gene expression regulation in *trans* (Rinn et al., 2007). Transcribed from the *HOXC* locus, *HOTAIR* was proposed to repress genes on the distal *HOXD* locus through the recruitment of the polycomb repressive complex 2 (PRC2) complex (Rinn et al., 2007). While subsequent studies have questioned both the role of *HOTAIR* in *HOXD* regulation (Amandio et al., 2016; Li et al., 2013; Schorderet & Duboule, 2011) and the specificity of the *HOTAIR*‐PRC2 interaction (Davidovich et al., 2013; Portoso et al., 2017), investigations of *HOTAIR* in the context of cancer have suggested an intriguing role in promoting metastatic progression through a related mechanism. Enforced expression of *HOTAIR* was shown to result in global redistribution of PRC2 occupancy and altered H3K27me3 patterns (Gupta et al., 2010). Thus, while the physiological functions and mechanisms of *HOTAIR* remain unclear, the potential role of overexpressed *HOTAIR* in causing widespread epigenetic alterations and gene expression changes might explain its role in promoting aggressive and invasive cellular phenotypes (Figure 5a). One might envision destabilizing *HOTAIR* or interfering with the *HOTAIR*‐PRC2 interaction as therapeutic strategies in *HOTAIR*‐overexpressing cancers.

The lncRNA *MALAT1* is similarly overexpressed in various cancer types, with increased *MALAT1* levels being highly predictive of poor patient prognosis (Gutschner, Hammerle, & Diederichs, 2013). These observations have suggested that overexpressed *MALAT1* may be a driver of cancer progression and that targeting *MALAT1* may be an exciting therapeutic strategy in patients with advanced disease (Amodio et al., 2018). Indeed, ASO gapmer‐mediated *MALAT1* knockdown was shown to be highly effective in decreasing tumor burden, suppressing metastatic dissemination, and increasing tumor differentiation in xenograft models of metastatic lung and breast adenocarcinomas (Arun et al., 2016; Gutschner et al., 2013). An independent strategy for inhibiting *MALAT1* has been the development of small molecules targeting a unique 3′ terminal triplex structure, called the ENE element, which is required for *MALAT1* stability (Brown et al., 2014; Wilusz et al., 2012). Treatment with ENE triplex‐binding chemotypes led to *MALAT1* destabilization and decreased morphogenesis of mammary tumor organoids (Abulwerdi et al., 2019). Interestingly, similar to *HOTAIR*, the physiological function of *MALAT1* is not well understood. *MALAT1* is ubiquitously and highly expressed in all normal cell types and has been shown to localize to nuclear speckles, which are nuclear structures implicated in gene expression and splicing control (Tripathi et al., 2010; West et al., 2014). However, three independent loss‐of‐function mouse models of *Malat1*—one with deletion of the *Malat1* promoter, one with a deletion encompassing the *Malat1* transcript, and one with insertion of a STOP cassette to terminate *Malat1* transcription—did not present with any overt phenotypes at the organismal levels and suggested limited and inconsistent alterations in gene expression and splicing patterns (Eissmann et al., 2012; Nakagawa et al., 2012; Zhang et al., 2012). It is conceivable that in the context of overexpression in cancer, *MALAT1* may acquire novel gain‐of‐function activities that lead to transcriptome deregulation and promote cancer progression (Figure 5b).

#### 3.1.2 Perturbation of cellular pathways by lncRNAs acting as molecular decoys

**FIGURE 6:**
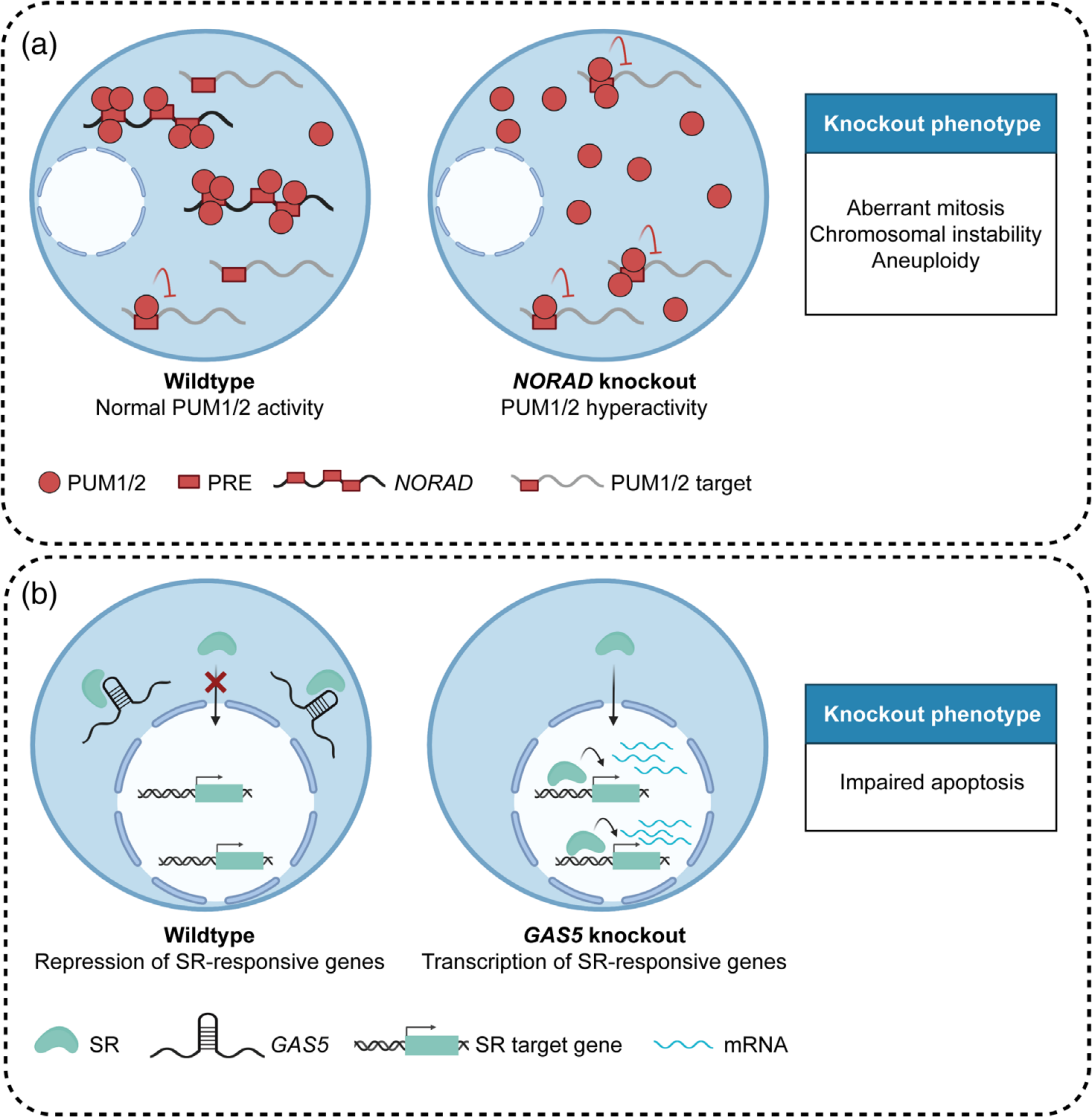
*Trans*‐acting lncRNAs act as decoys to fine‐tune cellular pathways. (a) *Left*—*NORAD* harbors 15 PREs that allow it to sequester PUM proteins and protect PUM target mRNAs from the inhibitory effects of PUM proteins (Lee et al., 2016). *Right*—Loss of *NORAD* leads to PUM target downregulation and results in a wide range of cellular phenotypes, including genomic instability; (b) *Left—*The predominantly cytoplasmic lncRNA *GAS5* binds SRs via a stem–loop structure that mimics genomic GREs and leads to decreased transcription of SR‐responsive genes. *Right—*Downregulation of *GAS5* in breast and prostate cancer results in increased transcription of SR target genes and resistance to apoptosis (Mourtada‐Maarabouni et al., 2009; Pickard et al., 2013)

One appealing mechanism has been the potential of lncRNAs to act as decoys to modulate the abundance and/or localization of binding partners. The PUM‐interacting lncRNA *NORAD* has provided an excellent example of this paradigm. PUM proteins are known to negatively regulate the stability and translation of a network of PRE‐containing mRNAs with diverse roles in cancer‐relevant processes, such as genome stability and differentiation (Smialek et al., 2021). In normal cells, PUM proteins and *NORAD* maintain a functional stoichiometry, which allows *NORAD* to sequester PUM proteins in NP bodies and negatively regulate the activities of PUM proteins (Elguindy & Mendell, 2021; Figure 6a). It remains to be determined how the altered levels of *NORAD*, observed across multiple cancer types, might affect PUM‐dependent processes and tumorigenesis (Soghli et al., 2021).

The abundant lncRNA growth arrest‐specific transcript 5 (*GAS5*) has similarly been reported to sequester and inhibit the activity of steroid receptors (SRs) through a conserved SR recognition sequence (Hudson et al., 2014; Kino et al., 2010). Upon agonist binding, SRs translocate to the nucleus to activate an elaborate network of downstream targets involved in cell survival, apoptosis, and metabolism. Consistent with *GAS5* being a repressor of SR signaling, *GAS5* is frequently downregulated in both breast and prostate cancer, suggesting a tumor‐suppressive role (Mourtada‐Maarabouni et al., 2009; Pickard et al., 2013; Figure 6b).

Analogous to *NORAD* and *GAS5*, several other lncRNAs have also been proposed to perturb cancer‐associated cellular pathways through specific interactions with protein binding partners. For example, overexpression of *Matar25* (mammary tumor‐associated RNA 25, human ortholog *LINC01271*) in breast cancer has been proposed to augment the activity of its interaction partners, PurA and PurB, which are transcriptional co‐activators required for the expression of key cancer genes, including the epithelial–mesenchymal transition factor *Tns1* (Tensin 1; Chang et al., 2020). Consequently, knockdown *Matar25* in cells and xenograft mouse models was found to lead to a notable reduction of cancer phenotypes, including metastatic dissemination to the lung (Chang et al., 2020). Analogously, pluripotency and hepatocyte‐associated RNA overexpressed in hepatocellular carcinoma (*PHAROH*) has been shown to regulate MYC protein levels by specifically recruiting and sequestering the translational repressor T‐cell restricted intracellular antigen‐related protein (TIAR; Yu et al., 2021). Another example is damage‐induced noncoding (*DINO*), a p53‐induced lncRNA expressed from the *Cdkn1a/p21* locus, similarly to *LincRNA‐p21*, but implicated as a *trans* amplifier of the p53 tumor suppressor pathway (Schmitt et al., 2016). *DINO* has been proposed to act by binding to and stabilizing p53 in a positive feed‐forward loop. As a result, loss of *DINO* expression through promoter hypermethylation, an epigenetic event mutually exclusive with TP53 alterations, was observed to impair the p53 signaling pathway and compromise p53 tumor suppressor function (Marney et al., 2021).

Finally, a substantial body of work has focused on the proposed role of lncRNAs in ceRNA (competing endogenous RNA) networks through direct base pairing and sequestration of miRNAs (microRNAs), leading to increased expression of miRNA target genes (Tay et al., 2014). This mechanism is theoretically viable, as illustrated by the well‐studied circular RNA (circRNA) *ciRS‐7*. This transcript harbors 73 imperfect binding sites for miR‐7, a miRNA that is frequently co‐expressed with *ciRS‐7* in the brain (Hansen et al., 2013). Although these sites bind both miR‐7 and Argonaute 2 (AGO2), *ciRS‐7* resists miRNA‐mediated cleavage and degradation (Hansen et al., 2013; Piwecka et al., 2017). This effect, which likely stems from the incomplete match between miR‐7 and its target sites, also protects miR‐7 from the deleterious consequences of trimming and tailing (de la Mata et al., 2015; Piwecka et al., 2017). Thus, *ciRS‐7* reduces the effective concentration of miR‐7 without triggering its degradation. In keeping with this, overexpression of *ciRS‐7* in colon cancer cells results in the upregulation of several validated miR‐7 targets (Weng et al., 2017).

Apart from ciRS‐7, the functional stoichiometry and significance of ceRNA networks remain to be validated. Many lncRNAs, pseudogenes, and circRNAs that harbor miRNA binding sites have been proposed to act through ceRNA mechanisms (Guo et al., 2014; Poliseno et al., 2010; Tang et al., 2016). However, many of these transcripts are lowly expressed and have no more miRNA binding sites than would be expected based on chance (Guo et al., 2014). Indeed, one study found that miRNA targets were derepressed only after the addition of over 150,000 target sites per cell, suggesting that most putative ceRNAs are not likely to contribute enough target sites to alter the expression of transcripts repressed by miRNAs (Denzler et al., 2014). An understanding of the ceRNA:miRNA:mRNA stoichiometry is thus a vital first step towards establishing the plausibility of a widespread ceRNA hypothesis for lncRNA activities.

#### 3.1.3 Annotated lncRNAs as sources of novel proteins

Finally, a series of recent studies have brought to attention the overlooked roles of small peptides encoded within putative lncRNAs. As an example, terminal differentiation‐induced ncRNA (*TINCR*) was initially proposed to enhance the stability of target mRNAs in *trans* by forming base‐pairing interactions with a 25‐nucleotide motif called the *TINCR* box (Kretz et al., 2013). However, this lncRNA was also found to harbor an ultraconserved open reading frame (ORF) encoding a small peptide, suggesting that *TINCR* transcripts may perform both coding‐dependent and coding‐independent functions (Eckhart et al., 2020). Given the frequent deregulation of *TINCR* in many types of cancer, it will be important to determine the relative contributions of its coding and noncoding elements. The long intergenic noncoding p53‐induced transcript (*LINC‐PINT*) has provided an analogous example. Initially implicated as a PRC2‐interacting lncRNA, later studies revealed the presence of a conserved, functional peptide produced from a circular isoform of *LINC‐PINT* (Marin‐Bejar et al., 2013; Marin‐Bejar et al., 2017; Zhang et al., 2018). In‐depth investigation of the taurine‐upregulated gene (*Tug1*) locus also revealed layers of functional elements, including a *cis*‐regulatory DNA sequence, a *trans‐*acting lncRNA, and an evolutionary conserved ORF, encoding a protein involved in mitochondrial membrane potential (Lewandowski et al., 2020). These examples have revealed the striking functional and mechanistic diversity and complexity of lncRNA loci and have highlighted the challenges in studying them (Housman & Ulitsky, 2016).

### 3.2 *Cis*‐acting lncRNAs in cancer

#### 3.2.1 Deregulation of dosage compensation and epigenetic unbalance

The *cis‐*acting lncRNA *XIST*, which interacts with a cohort of protein‐binding partners, including epigenetic regulators and nuclear scaffold proteins to enact X chromosome inactivation, has intrigued cancer researchers for decades (Brockdorff et al., 2020; Sahakyan et al., 2018). Early studies revealed that men with Klinefelter syndrome, who are characterized by an extra X chromosome, have an increased risk of many malignancies including breast cancer and non‐Hodgkin lymphoma (Swerdlow et al., 2005). Similarly, loss of X chromosome inactivation has been observed in breast cancer cell lines and testicular germ cell tumors (Kawakami et al., 2003; Sirchia et al., 2005). Beyond these correlative observations, a conditional *Xist* deletion model in mouse blood cell lineages resulted in widespread gene expression changes and led to aggressive myeloproliferative neoplasm and myelodysplastic syndrome with complete penetrance, likely due to global gene expression changes (Yildirim et al., 2013). This work revealed the important contributions of lncRNAs, such as *XIST*, in maintaining epigenetic balance. Further studies should determine the prevalence of *XIST* and X inactivation perturbations in human cancer and investigate the possibility of targeting this pathway as a therapeutic strategy.

Several lncRNAs involved in the local repression of imprinted loci also show significant deregulation in cancer, including *KCNQ1OT1*, *H19*, and maternally expressed gene 3 (*MEG3*), which are frequently overexpressed in a wide range of cancer types (O'Neill, 2005). In the case of *KCNQ1OT1*, its role in locally silencing the expression of the neighboring tumor suppressor cyclin‐dependent kinase inhibitor 1C (*CDKN1C*) may account for its oncogenic deregulation in cancers (Feng et al., 2018; Li et al., 2021; Wu et al., 2020). Additionally, inappropriate expression of the maternal *KCNQ1OT1* allele occurs in many cases of Beckwith–Wiedemann Syndrome, a condition associated with an increased risk of pediatric cancer (Diaz‐Meyer et al., 2003). A series of genetic experiments have revealed key functional elements within *KCNQ1OT1* and highlighted the importance of transcription as well as RNA‐mediated recruitment of chromatin‐modifying enzymes in mediating allele‐specific gene repression (Mancini‐DiNardo et al., 2006; Mohammad et al., 2008, 2010; Pandey et al., 2008; Schertzer et al., 2019). Two important unanswered questions are whether the cancer‐associated overexpression of *KCNQ1OT1* and other imprinted lncRNA arises from the imprinted allele, which normally does not express the lncRNA, and whether the overexpressed lncRNA has tumor‐promoting activities primarily through gene expression deregulation in *cis* or global transcriptome effects in *trans*.

#### 3.2.2 The complex relationship between MYC and surrounding lncRNAs

**FIGURE 7:**
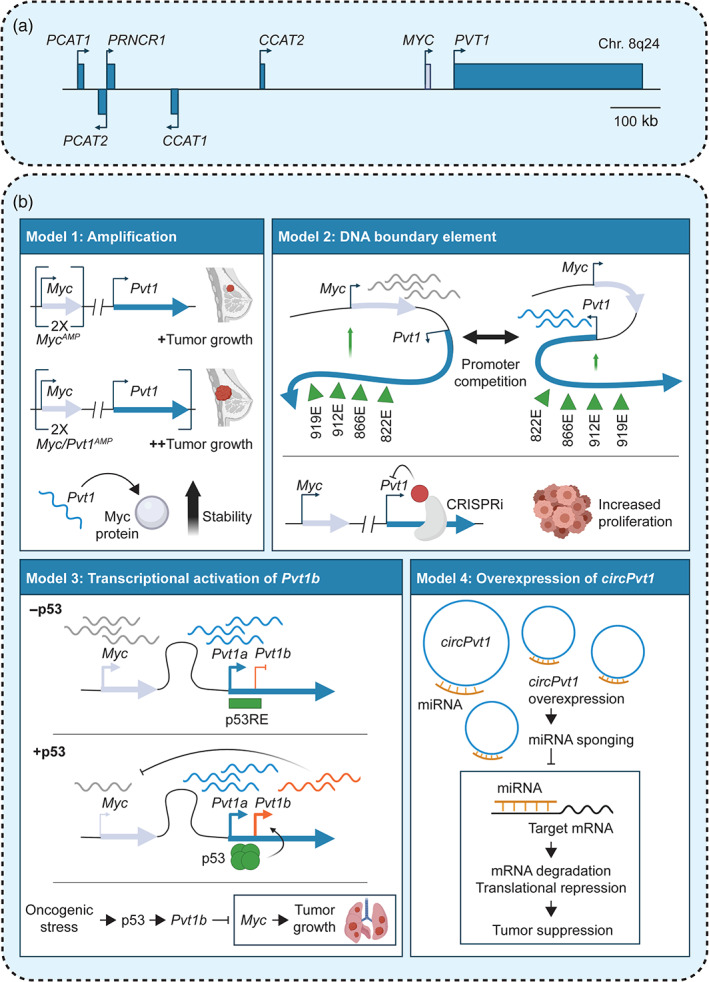
*Cis*‐regulatory lncRNAs expressed from the *Myc* locus. (a) The *Myc* locus harbors multiple lncRNAs that are subject to frequent genomic and epigenetic alteration in cancer; (b) *Top left*, *Model 1*: *PVT1* was proposed to act as an oncogene following the observation that amplification of the *Myc*/*Pvt1* locus in a mouse breast cancer model is more tumorigenic than amplification of the *Myc* locus alone (Tseng et al., 2014). Mechanistically, siRNA‐mediated knockdown of *Pvt1* led to decreased Myc protein levels, suggesting that *Pvt1* promotes Myc protein stability (Tseng et al., 2014). *Top right*, *Model 2*: An alternative model based on CRISPRi proposed that the *Pvt1* locus acts as a tumor‐suppressive DNA boundary element that limits access of *Myc* to *Pvt1* intragenic enhancers and suppresses *Myc* transcription (Cho et al., 2018). *Bottom left*, *Model 3*: The identification of a p53 response element (p53RE) within the *Pvt1* locus has led to the discovery of a p53‐dependent isoform of Pvt1, *Pvt1b* (Barsotti et al., 2012; Olivero et al., 2020; Porter et al., 2017). *Pvt1b* has emerged as a key mediator of p53 tumor‐suppressive function through its role in downregulating Myc and promoting senescence (Olivero et al., 2020; Tesfaye et al., 2021). *Bottom right*, *Model 4*: A circular isoform of *Pvt1*, *circPvt1*, has been proposed to play an oncogenic function through a putative ceRNA mechanism (Panda et al., 2017)

A 2 Mb segment mapping to human chromosome 8q24 and surrounding the MYC proto‐oncogene is a major hotspot of SNPs and large scale genomic alterations strongly associated with cancers of the breast, colon, head and neck, pancreas, ovaries, prostate, and bladder and highly predictive of poor patient outcome (Grisanzio & Freedman, 2010). Notably, beyond *MYC*, this region contains multiple lncRNAs, which are frequently the targets of genetic and epigenetic alterations and which have been implicated as positive and negative regulators of MYC (Huppi et al., 2012; Figure 7a).

In particular, the lncRNA *PVT1* has garnered significant attention because of its relationship with *MYC* (Colombo et al., 2015). *PVT1*, which is transcribed 52 kilobases downstream of *MYC*, is co‐amplified with *MYC* in many cancers and its increased expression is associated with a poor prognosis (Cui et al., 2016; Lu et al., 2017). An initial study modeled *Pvt1* and *Myc* co‐amplification in a series of elegant mouse models and confirmed a role for *Pvt1* amplification in promoting aggressing disease (Tseng et al., 2014; Figure 7b, Model 1). This study further proposed that the *Pvt1* lncRNA molecule acts in *trans* to stabilize the Myc protein (Tseng et al., 2014). However, subsequent work focused on the ability of the *PVT1* transcriptional unit to negatively regulate *MYC* in *cis*. The *PVT1* locus harbors multiple enhancers that engage in long‐range chromatin interactions with the *MYC* promoter (Fulco et al., 2016). In one study, the *PVT1* promoter—but not its RNA product—was found to act as a DNA boundary element that restricts access of the *MYC* promoter to *PVT1* intragenic enhancer (Cho et al., 2018; Figure 7b, Model 2). In contrast, several studies highlighted p53‐dependent repression of *Myc* through the transcriptional activation of an isoform of *Pvt1*, called *Pvt1b*, and determined the importance of this axis in mediating p53 tumor‐suppressive function (Barsotti et al., 2012; Olivero et al., 2020; Porter et al., 2017; Figure 7b, Model 3). Notably, evidence for putative tumor‐suppressive elements in the *PVT1* locus remains to be observed in human cancer, where *PVT1* is typically upregulated compared to normal cells. Finally, *circPVT1*, a circular isoform arising from the *PVT1* locus has been proposed to play an oncogenic function by suppressing cellular senescence and promoting proliferation through a ceRNA‐based mechanism (Panda et al., 2017; Figure 7b, Model 4). Given the diversity of proposed functional elements and mechanisms, additional studies are required to deconvolve the oncogenic and tumor‐suppressive elements in the *PVT1* locus.

Analogously to *PVT1*, altered expression and genetic variations in two lncRNAs upstream of *MYC*, colon cancer‐associated transcripts 1 and 2 (*CCAT1* and *CCAT2*), have also been correlated with increased susceptibility to colorectal cancer. As in the example above, it is not clear whether *CCAT1* (also known as *CARLo‐5*) and *CCAT2* act through DNA‐, RNA,‐ or transcription‐based mechanisms. Previous work has indicated that *CCAT1* facilitates long‐range interactions between *MYC* and an enhancer element, while *CCAT2* overlaps a putative *MYC* regulatory element (Jia et al., 2009; Kim et al., 2014; Pomerantz, Ahmadiyeh, et al., 2009; Pomerantz, Beckwith, et al., 2009; Xiang et al., 2014). To investigate the functional importance of the *CCAT2*‐associated element, genetically engineered mice lacking the ~2 kb‐long enhancer region were generated (Sur et al., 2012). Compared to controls, these animals were found to be markedly resistant to intestinal tumorigenesis in the context of the APC_min_ mouse model of colon cancer, indicating the key relevance of this element in colon cancer development (Sur et al., 2012). Subsequent studies have suggested that a specific SNP variation within this region may lead to increased *CCAT2* expression and greater predisposition to colorectal cancer (Ling et al., 2013). Future studies are needed to determine whether increased *CCAT*2 expression is the driver of the tumorigenic process and whether the *CCAT2* transcript itself or a DNA regulatory sequence from the region is responsible for the pro‐oncogenic effects.

Finally, a number of prostate cancer‐specific lncRNAs are also expressed from the 8q24 locus. These include prostate cancer‐associated noncoding RNA transcript 1 (*PCAT1*; Prensner et al., 2011), prostate cancer noncoding RNA 1 (*PRNCR1*; Chung et al., 2011; Yang et al., 2013), and prostate cancer‐associated noncoding RNA transcript 2 (*PCAT2*; Han et al., 2016). This set of lncRNAs has been found to be highly upregulated in prostate cancer and strongly predictive of poor patient outcomes. Accumulating evidence for long‐range chromatin interactions encompassing these lncRNAs and neighboring protein‐coding genes, including *MYC*, have suggested functional interconnections between the different elements in the locus but detailed mechanistic insights are missing (Cai et al., 2016; Jia et al., 2009). In particular, it remains to be determined whether or not this set of lncRNAs is directly involved in regulating *MYC* expression *in cis* or have primarily *MYC*‐independent functions.

#### 3.2.3 *Cis*‐acting lncRNA in the p53 tumor suppressor pathway

**FIGURE 8:**
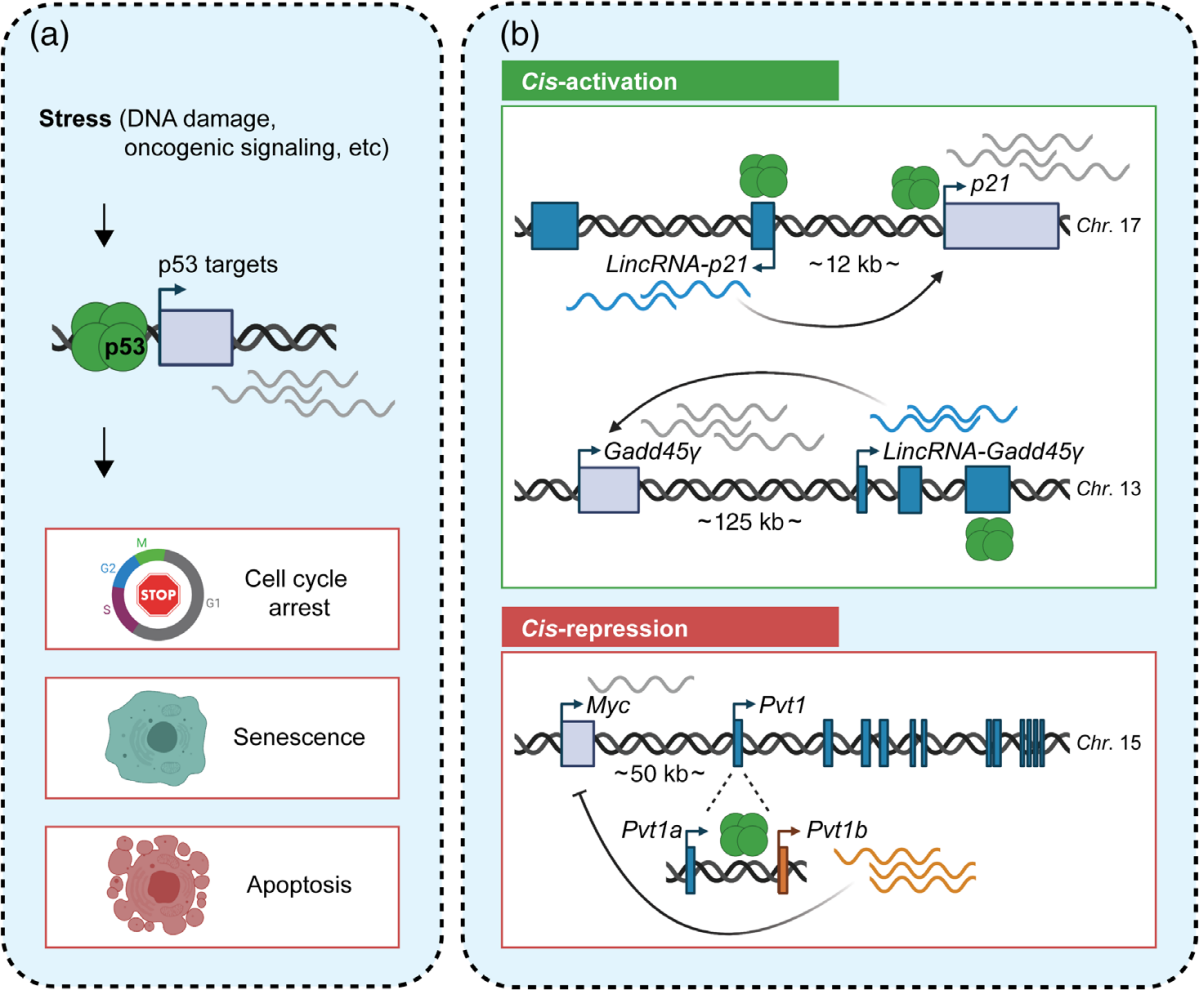
*Cis*‐regulatory lncRNAs are critical mediators of the p53 transcriptional network. (a) In the presence of stress, such as DNA damage or oncogenic signaling, the p53 tumor suppressor protein induces transcription of a broad network of target genes, including many lncRNAs. The cellular outcomes of p53 activation include cell cycle arrest, senescence, and apoptosis; (b) Many p53‐induced lncRNAs exhibit *cis*‐regulatory activities (Tesfaye et al., 2021). *Top—LincRNA‐p21*, which reinforces transcription of the nearby cell cycle checkpoint inhibitor *p21*, and *Linc‐Gadd45γ*, which induces expression of the noncanonical p53 target *Gadd45γ*, showcase the ability of some of these lncRNAs to promote local gene expression (Dimitrova et al., 2014; Tesfaye et al., 2021). *Bottom—*The lncRNA *Pvt1b* highlights the ability of *cis*‐regulatory lncRNAs to mediate local gene repression downstream of p53 (Olivero et al., 2020; Tesfaye et al., 2021). Figure not to scale

A recent characterization of the p53‐regulated transcriptome in response to oncogenic signaling revealed that as many as 30% of p53 target genes are lncRNAs (Tesfaye et al., 2021; Figure 8a). Interestingly, oncogenic stress‐induced lncRNAs predominantly showed chromatin association and appeared to have *cis*‐regulatory activities (Figure 8b). As an example, transcription of the novel lncRNA, *LincRNA‐Gadd45γ* was found to be both necessary and sufficient for activation of the neighboring cell cycle and apoptosis factor *Gadd45γ* (Tesfaye et al., 2021). These data are consistent with recent work on other p53‐regulated lncRNAs, including *LincRNA‐p21*, implicated in transcriptional activation of the neighboring cell cycle inhibitor *Cdkn1a/p21* past a functional threshold (Dimitrova et al., 2014), and *Pvt1b*, implicated in the transcriptional repression of the neighboring proto‐oncogene *Myc* (Olivero et al., 2020). These findings revealed that many lncRNAs in the p53 transcriptional network are involved in fine‐tuning local gene expression, although it remains to be established whether or not the mature RNA molecules play a role.

The functional importance of local gene regulation in the p53 tumor suppressor pathway has been highlighted by the *Pvt1* isoform *Pvt1b*, which relays the activating signal of p53 to a locally repressive signal to downregulate *Myc* transcription during the cellular response to stress (Olivero et al., 2020). Mutagenesis of the *Pvt1b*‐associated p53 binding site was shown to be sufficient to dramatically enhance early adenoma growth in an autochthonous mouse model of lung cancer. Strikingly, inhibition of *Pvt1b* resulted in an increase in tumor burden that was comparable to the burden observed in p53‐deficient tumors (Olivero et al., 2020). Mechanistically, *Pvt1b* suppressed tumor growth by promoting cellular senescence (Tesfaye et al., 2021). It remains to be determined how *cis*‐regulatory lncRNA loci, such as the *Pvt1b/Myc* locus, may be manipulated for therapeutic purposes.

#### 3.2.4 Antisense transcripts

The antisense lncRNAs antisense noncoding RNA in the *INK4* locus (*ANRIL*) and antisense noncoding *RASSF1* (*ANRASSF1*) have been implicated in the pathological repression of overlapping tumor suppressor genes. Transcription of *ANRIL* from the *INK4B/ARF/INK4A* locus correlates with the silencing of the cell cycle regulators *INK4A* and *INK4B*, thus accounting for the observation that high *ANRIL* levels are typically associated with aggressive disease progression and poor patient survival (Kong et al., 2018; Kotake et al., 2011; Yap et al., 2010; Yu et al., 2008). Similarly, *ANRASSF1*, which is frequently overexpressed in breast and prostate cancer, has been proposed to downregulate the expression of the overlapping *RASSF1A*, a tumor suppressor factor involved in cell cycle and apoptosis control (Beckedorff et al., 2013). *ANRIL* and *ANRASSF1* have both been proposed to mediate gene silencing through their association with PRC2 proteins. Since PRC2 exhibits widespread sequence‐independent interactions with lncRNAs, it is unclear whether transcription through the *ANRIL* and *ANRASSF1* loci is sufficient for local gene repression (Davidovich & Cech, 2015).

#### 3.2.5 Transcribed DNA elements

The genome harbors myriad *cis*‐regulatory DNA elements that are characterized by low nucleosome occupancy and abundance of transcription factor binding sites. The observation that Pol II readily initiates transcription from regions of open chromatin has given rise to the hypothesis that many noncoding transcriptional events may represent noise and generate nonfunctional “junk” RNAs (Espinosa, 2016). Under this paradigm, the DNA sequence elements within a lncRNA‐generating locus may be both necessary and sufficient for local regulatory activity, while the transcriptional process and mature RNA molecule are non‐functional byproducts. This model has been illustrated by the lncRNA downstream of *Cdkn1b* (*LockD*) locus, where regional deletion, but not PAS insertion, affected the expression of the neighboring gene (Paralkar et al., 2016). Conversely, it has been proposed that the act of transcription may play important role in promoting chromatin organization and nuclear architecture (Mele & Rinn, 2016).

## 4 CONCLUSION

A growing body of work has linked alterations in lncRNAs to cancer initiation and progression. Additionally, an array of complementary genetic, molecular, and biochemical approaches has been employed in an effort to elucidate the functions and mechanisms by which these lncRNAs contribute to tumorigenesis. These studies have provided intriguing examples of how alterations in lncRNAs result in the sequestration or redistribution of lncRNA‐binding partners and lead to perturbations of the epigenetic state, gene expression balance, or other processes in cancer cells. These types of gain‐of‐function activities have emerged as the predominant mechanism by which overexpressed *trans*‐acting lncRNAs perturb cellular homeostasis and enable the acquisition of cancer hallmarks. A similar pattern has also been observed for *cis*‐acting lncRNAs. On the one hand, low abundance, *cis*‐acting lncRNAs have been found to mislocalize to distant sites in the context of oncogenic overexpression, leading to gain‐of‐function *trans* activities. As an example, *LincRNA‐p21*, which is chromatin‐associated and expressed at just a few copies per cell, appears to be highly overexpressed and to localize and function in the cytoplasm of several human cancer cell lines (Yang et al., 2014; Yoon et al., 2012). On the other hand, the oncogenic upregulation or downregulation of high abundance, *cis*‐acting lncRNAs, such as *XIST*, may also result in the sequestration or release, respectively, of a large pool of protein‐binding factors, including chromatin modifiers and transcriptional regulators, which may in turn lead to a global epigenetic unbalance. There have also been examples of how perturbations in *cis*‐regulation can contribute to cellular transformation through local disruption of gene expression patterns. Taking all of these examples into account, the emerging theme is that deregulated lncRNAs frequently acquire gain‐of‐function activities that may or may not be related to their physiological functions. Thus, lncRNA‐targeting therapies focused on lncRNA degradation or on the disruption of functional interactions may be effective in reversing cancer phenotypes.

Finally, it is important to note that, in addition to RNA‐based models, the functional impact of unconventional mechanisms should also be considered. The growing number of short functional peptides encoded by putative *trans*‐acting lncRNAs has opened new avenues for exploration. Similarly, elucidating the local interplay between regulatory DNA elements, the act of transcription, and the functional elements in the mature lncRNA molecule will be essential to dissecting the mechanisms of *cis*‐regulatory loci.

## CONFLICT OF INTEREST

The authors declare no conflicts of interest.

## AUTHOR CONTRIBUTIONS

**Lauren Winkler:** Conceptualization (equal); writing – original draft (lead); writing – review and editing (equal). **Nadya Dimitrova:** Conceptualization (equal); funding acquisition (lead); writing – original draft (supporting); writing – review and editing (equal).

## RELATED WIREs ARTICLE

Cytoplasmic functions of long noncoding RNAs

## Data Availability

Data sharing is not applicable to this article as no new data were created or analyzed in this study.
